# The Role of Antibodies in the Pathogenesis of Multiple Sclerosis

**DOI:** 10.3389/fneur.2020.533388

**Published:** 2020-10-20

**Authors:** Xiaoli Yu, Michael Graner, Peter G. E. Kennedy, Yiting Liu

**Affiliations:** ^1^Department of Neurosurgery, University of Colorado Anschutz Medical Campus, Aurora, CO, United States; ^2^Institute of Infection, Immunity and Inflammation, University of Glasgow, Glasgow, United Kingdom; ^3^Department of Neurology, University of Colorado Anschutz Medical Campus, Aurora, CO, United States

**Keywords:** multiple sclerosis, antibody, oligoclonal bands, immunoglobulin G, cytotoxicity, cerebrospinal fluid, serum, B cells

## Abstract

The presence of persistent intrathecal oligoclonal immunoglobulin G (IgG) bands (OCBs) and lesional IgG deposition are seminal features of multiple sclerosis (MS) disease pathology. Despite extensive investigations, the role of antibodies, the products of mature CD19^+^ B cells, in disease development is still controversial and under significant debate. Recent success of B cell depletion therapies has revealed that CD20^+^ B cells contribute to MS pathogenesis via both antigen-presentation and T-cell-regulation. However, the limited efficacy of CD20^+^ B cell depletion therapies for the treatment of progressive MS indicates that additional mechanisms are involved. In this review, we present findings suggesting a potential pathological role for increased intrathecal IgGs, the relation of circulating antibodies to intrathecal IgGs, and the selective elevation of IgG1 and IgG3 subclasses in MS. We propose a working hypothesis that circulating B cells and antibodies contribute significantly to intrathecal IgGs, thereby exerting primary and pathogenic effects in MS development. Increased levels of IgG1 and IgG3 antibodies induce potent antibody-mediated cytotoxicity to central nervous system (CNS) cells and/or reduce the threshold required for antigen-driven antibody clustering leading to optimal activation of immune responses. Direct proof of the pathogenic roles of antibodies in MS may provide opportunities for novel blood biomarker identification as well as strategies for the development of effective therapeutic interventions.

## Introduction

Multiple sclerosis (MS) is a chronic inflammatory disease that affects the central nervous system (CNS), especially the brain, spinal cord, and optic nerves. About 1 million people in the US and 2.5 million worldwide live with a diagnosis of MS (1). After its first description in 1868 by Jean-Martin Charcot, MS has been classified into different types such as clinically isolated syndrome (CIS, a clinical syndrome highly suggestive of a first manifestation of MS), relapsing remitting (RR), secondary progressive (SP), and primary progressive (PP) (2). Extensive pathological studies have classified MS lesions as active, chronic active, inactive, and pre-active stages (3). Despite the heterogeneous features of MS lesions, a consensus has emerged that the pathogenic mechanisms of the disease are contributed by CNS inflammation and infiltration of peripheral immune cells, resulting in neuronal and glial cell injury and subsequent loss of myelin sheath around nerves, interruption of axonal communication, and neurologic deficits (4). However, the exact cause of MS is unknown, and currently there is no cure for the disease.

The presence of persistent CNS oligoclonal immunoglobulin G (IgG) bands (OCBs) and lesional IgG deposition are hallmarks of MS. OCBs consist of clonally restricted immunoglobulins detected by isoelectric focusing (IEF) and are a key feature of ongoing inflammatory events in CNS in a number of neuro-inflammatory conditions and viral infections (5). Although the pathological effects of OCBs have been implicated since their discovery (6), the role of antibodies in the pathogenesis of MS is controversial. In this review, we discuss pathological and immunological studies regarding the role of antibodies in MS. We propose a novel framework regarding the pathogenic mechanism of disease, which could be mediated by increased levels of serum IgG1 and IgG3 antibodies.

## Increased Intrathecal Synthesis of OCB is the Most Characteristic Feature of MS

Early biochemical studies of MS autopsy brain plaques with active lesions have demonstrated the presence of excessive amounts of IgG antibodies in both free/soluble and tissue-bound/particulate forms (7–9). The IgGs extracted from corresponding soluble and particulate samples displayed OCBs (9). Extensive pathological characterization of heterogeneous MS autopsy brain samples has demonstrated the co-localization of IgG antibodies, complement, and Fc gamma receptors (FcγR) in the active lesions, suggesting a role for these antibodies in the early stages of the disease (10, 11). Furthermore, complement activation is found in PPMS cortical gray matter lesions (12), indicating that antibodies may contribute to the worsening pathology that underlies the irreversible progression of MS. These lines of evidence suggest that the excessive presence of IgG antibodies in MS lesions may induce complement-mediated and immune-cell-mediated cytotoxicity, resulting in lesion formation.

The single most consistent laboratory abnormality in MS is the presence of OCBs in the cerebrospinal fluid (CSF) of up to 95% of patients (13, 14). Once present, the pattern of OCB is characteristic for each individual and does not change within patients over years, despite therapeutic interventions (5, 13, 15, 16). Besides OCBs, the intrathecal IgG can also be visualized with Reiber diagram, which uses CSF/serum quotient diagrams with hyperbolic discrimination lines for IgG (17). Plasma cells are found in the chronically inflamed MS CNS (18). Long-lived plasma cells were demonstrated in chronically inflamed CNS and chemokine CXCL12 is involved in plasma cell persistence (19). For detailed review regarding the complex nature of resident plasma cells and mechanism driving the persistence in CNS, please see the review by Pryce and Baker (5). Using a phage-displayed random peptide library approach, we demonstrated that CSF IgGs obtained longitudinally from MS patients recognized identical epitopes over time, supporting the notion of a temporal stability of CSF IgG specificity (20).

Accumulating evidence supports the pathological role of CSF immunoglobulins. CSF OCBs were found to be associated with increased levels of disease activity and disability, with the conversion from a CIS to early RRMS, with greater brain atrophy, and with increased levels of disease activity (21–28). Further, CSF of MS patients induced inflammatory demyelination and axonal damage in mice (29, 30). We demonstrated that a subset of myelin-specific recombinant antibodies constructed from clonal expanded plasma cells in MS CSF caused robust complement-dependent cytotoxicity in oligodendrocytes and induced rapid demyelination in mouse organotypic cerebellar slices (31, 32). These studies support the pathogenic effects of CSF IgGs in MS. Despite the significance of OCB in MS, no statistically significant differences of both number of OCBs and IgG index were found among subtypes of MS (CIS, RRMS, PPMS, and SPMS) (33). New technologies such as recombinant antibodies generated from clonally expanded single B cells/plasma cells and directly from IgG sequences of OCBs provided promises for determining the specificities of OCB, but have so far failed to reveal a common targets of MS (34–37) (https://www.jni-journal.com/article/S0165-5728(20)30298-8/pdf).

Except for myelin, convincing CNS target antigens for OCBs that are specific to MS are not known. Recently, it was shown that some OCBs targeted ubiquitous self-proteins and intracellular antigens (37–39), suggesting that CSF antibodies may develop as a passive response to CNS injury, rather than mediating primary pathogenic effects. Besides MS, CSF OCBs have been reported in a number of neuro-inflammatory conditions and viral infections (40). It has been argued that this intrathecal, poly-specific, and oligoclonal immune response possibly indicates that it is not a specific antigen that drives the development of OCBs in MS, but rather a non-specific activation of CSF-localized B cells (41).

## The Sources of Increased Intrathecal IgG, a Controversy In MS

### Correlation of Serum Antibodies With Intrathecal IgGs

OCBs are thought to be produced by intrathecal parenchymal B lymphocytes, as the CSF Ig proteome and transcriptome of CSF-located B cells matched each other. In addition, intrathecal B cells show signs of somatic hyper-mutation and clonal expansion, pointing toward a germinal center-like reaction with antigen-driven affinity maturation within the CNS (42, 43). However, there is new evidence that terminally differentiated B cells in MS CSF are not solely derived from intrathecal maturation, but can emerge from the CNS compartment and interact with the peripheral immune system (44–46). Recent deep-immune repertoire studies revealed that MS CSF OCBs were not merely produced by CNS B cells, and some OCB specificities were related only to peripheral B cells, which indicate that disease-relevant B cells circulate between the CNS and peripheral compartments (47). We recently demonstrated that serum IgG in MS was significantly elevated and there was a strong correlation between CSF IgG and CSF albumin, and also between CSF IgG and serum IgG (48). Since CSF albumin is exclusively derived from the blood in MS, this correlation suggests that most of the CSF IgG is derived from the blood. It has to be noted that about 50% of MS sera did not show OCBs and patients with OCBs in sera had patterns either partially similar or completely different from those seen in matching CSF (49). Because serum IgG is about 200 times more concentrated than CSF IgG (48), it is possible that serum OCBs are masked by massive amounts of polyclonal IgGs.

Another line of evidence supporting serum and intrathecal IgG exchange comes from the discrepancy between number of CNS B cells and the quantities of intrathecal IgG. A careful examination of MS plaques concluded that there were far too few cells in the plaques to contribute to IgG (50). As calculated by Tourtellotte's formula, the normal values of the CNS IgG synthesis rate were lower than 3.3 mg/day and the median value in MS patients was 29 mg/day (51). It would take 3.2 billion lymphocytes in MS to generate such large amounts of CNS IgG (30 mg in 500 ml CSF) (52). We and others have demonstrated that CSF leukocyte counts in most MS patients were <50 cells/μl (about 2.5 million cells in 500 ml CSF), of which 5% were B cells (48, 53, 54). Therefore, CSF lymphocytes could only account for <0.1% of the IgG in the MS CSF per day. The low number of lymphocytes in MS CSF and the high level of intrathecal IgG raise the question as to whether CNS B cells in MS can be responsible for the massive amounts of elevated intrathecal IgG. This apparent knowledge gap suggests that most of the intrathecal IgG in MS may in fact be derived from the blood.

### MRI Detection of the Central Vein Sign in MS Lesions Supports a Peripheral Blood Contribution to Disease Activity

Early histopathological studies detected a unique character of MS lesions described as “centrifugally-spreading” that an MS plaque did not spread or grow at its edges. Plaques began as collars of demyelination around small veins and enlarged thereafter (50). This perivenous distribution of MS plaques was confirmed by ultra-high-field magnetic resonance imaging (MRI) (55–57). The MRI-detectable central vein sign inside white matter lesions can distinguish MS from other CNS inflammatory disorders and has been proposed as a biomarker for inflammatory demyelination (58). Further, the serum protein fibrinogen was found frequently and extensively to be present diffusely in both extracellular and intracellular spaces of MS motor cortex and in close proximity to blood vessels, and was related to the extent of neurodegeneration in progressive forms of MS (59). This line of evidence supports the notion of peripheral blood contribution of B cells and antibodies to intrathecal IgGs and their potential role in disease development.

**Figure 1:**
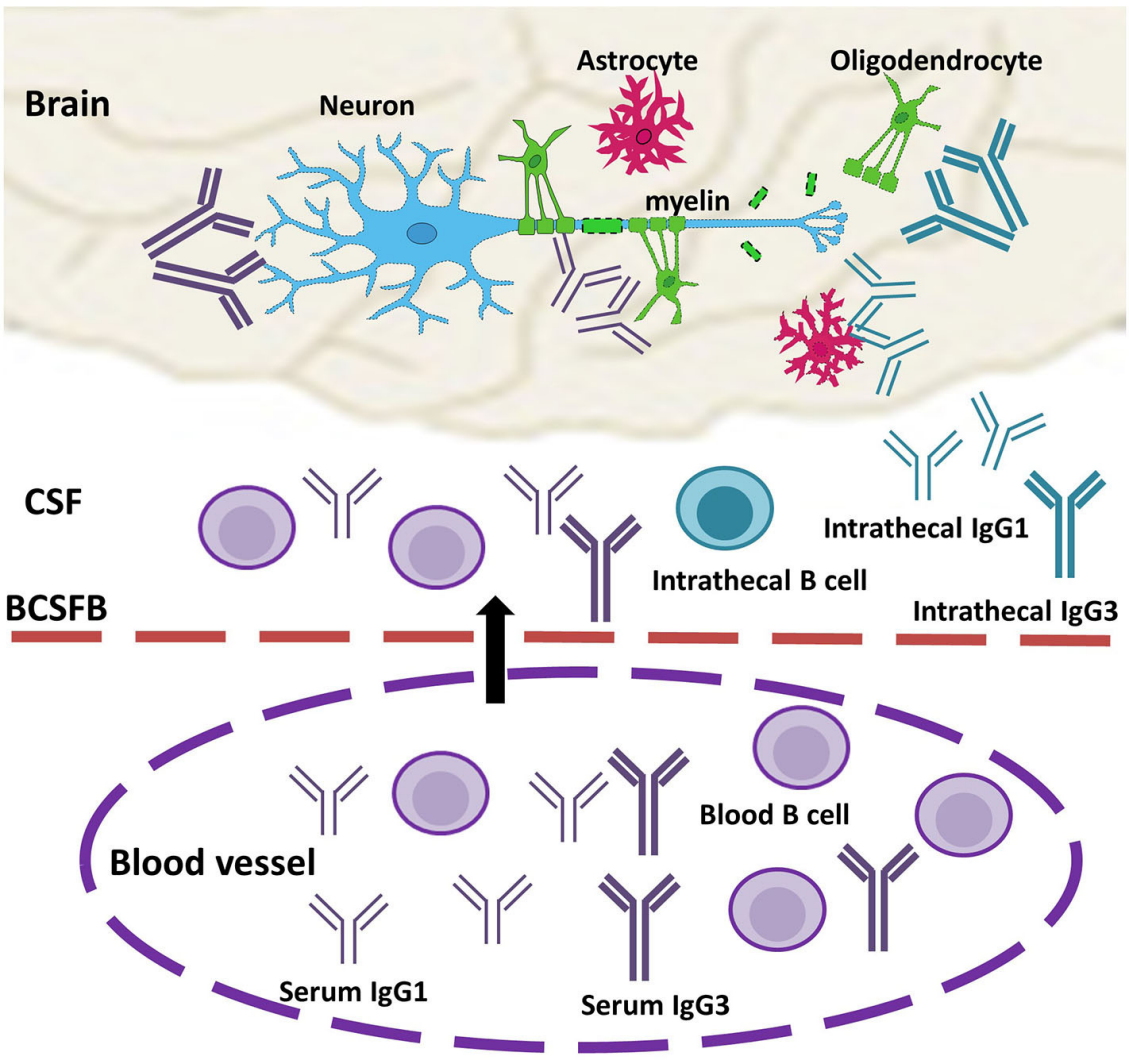
Model of the role of serum antibodies in MS disease pathogenesis. Circulating serum antibodies (IgG1 and IgG3, purple) and antibody-producing B cells migrate across the impaired blood-barrier (arrow), and they are present in CSF OCBs and CNS lesion together with intrathecal IgGs (IgG1 and IgG3, turquoise). In the brain, IgGs recognize antigens on the cell surfaces of neurons or/and glial cells and form immune complexes with complement factors and/or immune cells. Elevated levels of IgG1 and IgG3 induce enhanced cytotoxicity or reduced threshold to trigger injury response to CNS cells, which, in turn, result in loss of myelin sheath outside of axons. BCSFB, blood-CSF barrier.

We propose that MS intrathecal IgGs are derived from B cells in both the CNS and peripheral blood and may thus be contributed by serum antibodies (Figure 1). Inside the CNS compartments, clonally expanded antigen-experienced plasma cells produce antibodies that may target cell surface antigens and exert a pathogenic effect by activating complement-dependent or immune-cell dependent cytotoxicity. A subpopulation of these antibodies may direct against intracellular autoantigens released during tissue destruction. On the other hand, serum B cells and antibodies migrate across the blood barriers either by active transportation or by barrier breakdown. Some of these serum antibodies in MS are clearly pathogenic as reviewed below.

## Circulating Antibodies Contribute to MS Disease Pathogenesis

### Pathogenic Effect of Serum Antibodies

The benefit shown in therapeutic plasma exchange and immune-adsorption therapy in some MS patients (41, 60, 61) suggests that serum antibodies in MS are pathogenic. Patients who had lesions with prominent Ig deposition and complement activation profited most from plasma exchange (60, 62). However, direct proof of the pathogenic role of serum antibody in MS is complicated by the marked heterogeneity of the disease and the variability of experimental procedures.

Early *in vitro* demyelination studies have provided evidence supporting a pathogenic role of serum antibodies (50) and that there is strong correlation between disease activity and demyelinative activity of MS serum. Lumsden (50) investigated sera from 450 MS patients and controls over 7 years with 1,300 tests. He found that over 80% of MS sera with natural complement produced demyelination in live cultures of newborn rat cerebellum. Further, he found patients' immunoglobulins and complement were fixed anti-mortem to CNS components, indicating that circulating antibody in MS binds to myelin and causes demyelination. Lumsden's data indicate that serum antibodies in their natural state are pathogenic when they penetrate the CNS parenchyma. Using *ex vivo* assays, a number of laboratories have reported that some MS patients have serum factors that demyelinate myelinating explants (63–66). Later, complement-dependent demyelinating IgG response was detected with purified serum IgGs in ~30% of 37 MS patients (67). However, the demyelinating effect of MS serum IgGs has been controversial, which is due in part to variable tissue culture and myelin imaging methods. Additionally, we postulate that the different results may reflect the sources of antibodies used. The antibody purification procedures may result in loss of the natural state of antibodies and may fail to efficiently recover specific IgG subclasses, resulting in a substantial reduction or a complete loss of demyelinating effect. Lumsden's work was carried out using unpurified native serum antibodies (50).

The potential pathogenic role of serum antibodies may also extend to enhancing inflammatory responses across the BBB in MS. For example, significantly higher levels of anti-endothelial cell antibodies and immune complexes were found in MS sera (68), and serum antibodies from MS patients were detected in micro-vessels in brain tissues and bound to endothelia cells (69, 70). Further, sera from RRMS and SPMS disrupt the BBB (71). In summary, over 50 years of extensive scientific investigations have provided accumulating evidence that serum antibodies in their natural state exert primary antibody-dependent cytotoxicity to glial cells, which leads to demyelinating effects that could contribute to MS disease pathogenesis.

### Insights From B Cell Depletion Therapies

B cell depletion therapies using monoclonal antibodies against CD20; namely, Rituximab, Ocrelizumab, and Ofatumumab have shown profound success in controlling MS relapses (41). CD20 is a four-transmembrane protein expressed on the surface of B cells from the late pro-B cells through the memory cell stages, but not on antibody-producing plasma cells. Thus, the efficacy of this B cell depletion therapy has been considered to be mediated by B cell function independent of antibody production, such as antigen-presentation for the activation of T cells and pro-inflammatory cytokine secretion (72). Indeed, serum antibody level and CSF OCB often persist despite CD20-antibody depleting B cells (5, 73). In some instances, certain serum antibodies were reduced in a proportion of patients after intravenous Rituximab treatment (74). CSF plasma cell depletion was observed following repeated intrathecal Rituximab injection (75, 76). These reductions are thought to result from secondary effects such as depletion of plasma cell precursors, depletion of survival factors, or possibly destruction of B cell niches rather than a direct influence on plasma cells. Interestingly, Laquinimod, a T cell targeting oral disease-modifying therapy, has been shown to modulate myelin antigen-specific B cell immune response and inhibit development of MOG-specific IgG antibodies (77). And treatment of Cladribine (another T cell targeted drug) in MS is associated with depletion of memory B cells (78).

However, reports of MS patients' failure to respond to anti-CD20 therapies, or even disease exacerbation thereafter, have also been published. Anti-CD20 therapies have limited efficacy in inhibiting disease progression (41). It is possible that these therapies do not effectively target the antibody-producing B cells, or do not significantly reduce the antibody levels in serum and CSF. Another issue concerns the increased risk of infection that is likely to accumulate with continuous B cell depletion with time. In MS, only a fraction of B cells and antibodies are pathogenic, while other subsets of B cells and antibodies exert essential regulatory functions to limit chronic inflammation. For in-depth reviews regarding B cell therapies and B cell biology between subtypes of MS, please see review papers by Gelfand et al. (79), Fraussen et al. (80), and Myhr et al. (81). It will be very important to develop innovative strategies selectively abrogating pathogenic B cells and antibodies. Thus, identifying the specific features of pathogenic antibodies in MS is crucial for the development of successful therapeutic interventions.

## Elevated Levels of IgG1 and IgG3 Antibodies in CSF And Serum, An MS Specific Feature

### Selective Elevation of IgG1 and IgG3 in MS

The glycoprotein IgG can be separated into four subclasses: IgG1 (60–70% in plasma), IgG2 (20–30%), IgG3 (5–8%), and IgG4 (1–3%) (82). A selective elevation of the IgG1 in MS CSF was observed (83, 84). The elevation of IgG1 and IgG3 indices in MS were found more frequently than the elevation of the general IgG index (21). Patients with a relapse were significantly more frequently seropositive for anti-MOG and anti-MBP IgG3 than those in remission (85). Further, the IgG3 allotype G3m was MS-specific and present in active brain plaques (86). Subsequent studies demonstrated that the susceptibility to MS was associated with an IgG3 restriction fragment length polymorphism (87), and a GWAS study showed that intrathecal IgG synthesis in MS was significantly associated with the intronic region of the IgG3 heavy chain gene SNPs (88). The significance of IgG3 in MS was recently highlighted by a findings that higher serum IgG3 levels may predict the development of MS from CIS (89) and IgG3 + B cells are associated with the development of MS (90). These data suggest that the presence of higher levels of IgG1 and IgG3 antibodies may play a significant role in MS disease activity.

### Increased IgG1 and IgG3 Enhances Effector Functions

IgG3 has an extended hinge region with highest flexibility compared to other antibody subclasses. This subclass can probe less exposed antigens. This feature could contribute to the higher potential of IgG3, followed by IgG1, to antibody oligomerization and activation of effector functions, including enhanced antibody-mediated cellular cytotoxicity (ADCC); opsonophagocytosis; complement activation; and neutralization. Additionally, IgG3 has superior affinity to FcγR and the first component of complement cascade, C1q (78). Thus, increased IgG1 and IgG3 in MS serum and CSF may enhance immune-mediated cytotoxicity to CNS cells or may reduce the thresholds for antigen-driven antibody clustering for optimal activation of immune responses.

## Conclusions

The role of antibodies in MS disease mechanisms has been disputed over several decades due to the lack of direct and reproducible proof of pathogenic effects. The limited efficacy of CD20-B cell therapies in progressive MS patients and in controlling disease progression implicates that CD20-negative antibody-producing B cells, as well as antibodies, play an important role in disease pathogenesis. The combined accumulating data that there is a strong correlation between serum and CSF IgG, insufficient B cells to produce large quantities of intrathecal IgG, MRI detection of the central vein sign in MS, and presence of pathogenic serum antibodies provide evidence for the hypothesis that circulating antibodies contribute to increased intrathecal IgG synthesis in MS. We further propose that (1) serum antibodies exert primary and pathogenic effects in MS development; (2) increased IgG1 and IgG3 result in enhanced cytotoxicity to CNS cells and produce antibody-mediated injury in MS pathogenesis (Figure 1). This novel hypothesis may help to resolve the current controversy regarding the roles of antibodies in MS and may draw attention to the possibly pathogenic role of IgG3. It may also provide novel opportunities for blood biomarker identification and the development of effective therapeutic interventions for MS.

## Author Contributions

XY and YL wrote and edited the manuscript. PK and MG critically reviewed and edited the manuscript. All authors contributed to the manuscript.

## Conflict of Interest

The authors declare that the research was conducted in the absence of any commercial or financial relationships that could be construed as a potential conflict of interest.
